# Teaching Semantic Radicals Facilitates Inferring New Character Meaning in Sentence Reading for Nonnative Chinese Speakers

**DOI:** 10.3389/fpsyg.2017.01846

**Published:** 2017-10-23

**Authors:** Thi Phuong Nguyen, Jie Zhang, Hong Li, Xinchun Wu, Yahua Cheng

**Affiliations:** ^1^Beijing Key Laboratory of Applied Experimental Psychology, National Demonstration Center for Experimental Psychology Education, Faculty of Psychology, Beijing Normal University, Beijing, China; ^2^Department of Curriculum and Instruction, University of Houston, Houston, TX, United States; ^3^Department of Psychology, Ningbo University, Zhejiang, China

**Keywords:** semantic radical awareness, semantic radical teaching, transfer in language learning, lexical inference, Chinese as a foreign language (CFL)

## Abstract

This study investigates the effects of teaching semantic radicals in inferring the meanings of unfamiliar characters among nonnative Chinese speakers. A total of 54 undergraduates majoring in Chinese Language from a university in Hanoi, Vietnam, who had 1 year of learning experience in Chinese were assigned to two experimental groups that received instructional intervention, called “old-for-new” semantic radical teaching, through two counterbalanced sets of semantic radicals, with one control group. All of the students completed pre- and post-tests of a sentence cloze task where they were required to choose an appropriate character that fit the sentence context among four options. The four options shared the same phonetic radicals but had different semantic radicals. The results showed that the pre-test and post-test score increases were significant for the experimental groups, but not for the control group. Most importantly, the experimental groups successfully transferred the semantic radical strategy to figure out the meanings of unfamiliar characters containing semantic radicals that had not been taught. The results demonstrate the effectiveness of teaching semantic radicals for lexical inference in sentence reading for nonnative speakers, and highlight the ability of transfer learning to acquire semantic categories of sub-lexical units (semantic radicals) in Chinese characters among foreign language learners.

## Introduction

Chinese language is often described as having a logographic writing system and is well-known for the visual complexity of its characters. Because of its complex orthographic configuration, mastering thousands of Chinese characters becomes a great challenge for Chinese as a Foreign Language (CFL) learners, particularly for foreign students whose first language is alphabetic (Shi and Wan, 1998; Shen, 2005). Semantic radicals, which represent the semantic category information of Chinese characters, play an important role in character decoding and reading for both native and nonnative Chinese speakers (e.g., Chan and Nunes, 1998; Feldman and Siok, 1999; Shen and Ke, 2007; Williams and Bever, 2010; Tong and Yip, 2015; Wang et al., 2015).

Previous research has suggested the effectiveness of the explicit instruction of semantic radicals for young Chinese children's literacy development (Packard et al., 2006; Wu et al., 2009), and radical teaching is an effective strategy for learning Chinese characters using multimedia instructional software in nonnative speakers (Jin, 2003; Hao et al., 2010; Chen et al., 2013). However, semantic radical strategy is regarded as a strategy in which learners can use meaning cues of semantic radicals in Chinese character recognition (Zhao and Jiang, 2002), whether teaching semantic radicals would help readers infer the meanings of new characters using a semantic radical strategy in reading context for CFL learners is largely unknown. More interestingly, whether explicit teaching of semantic radicals would help CFL learners transfer the semantic radical strategy to figure out the meanings of unknown characters that contain new semantic radicals in sentence context is less understood. Understanding this lexical inference process in CFL learners has implication for effective instruction in bridging character learning and reading comprehension in Chinese. The current study aims to explore whether a short and intensive morphology-based instruction on semantic radicals enhances students' ability to infer new character meanings in a sentence-reading context for CFL learners represented by a homogeneous sample of Vietnamese students.

### Role of semantic radical in learning chinese word reading

Characters consisting of different strokes and stroke patterns (components or radicals, e.g., “口”, “十”), are the basic orthographic units in Chinese. Foreign students in the initial stage of Chinese learning tend to imagine a whole character's shape as a picture and try to memorize it mechanically (Shi and Wan, 1998; Jiang and Zhao, 2001; Zhao and Jiang, 2002). Logographic Chinese characters are often expected as pictures for the existence of simple Chinese characters that convey meaning through pictographic or ideographic representation (Ho et al., 2003). Pictographic characters directly depict the shapes of things, and ideographic characters represent abstract concepts. For instance, the pictographic character “木, /mu4/” is the drawing of a tree, and the ideographic character “本, /ben3/” points to the root of the tree (Li F., 2005). However, these single characters occupy a very small percentage of Chinese. There are only 364 pictographic characters and 125 ideographic characters among the thousands of characters (Li F., 2005). More than 80% of Chinese characters are semantic-phonetic compound (SPC) characters consisting of a semantic radical that serves the semantic category or the related meaning of the whole character (e.g., “日” means *the sun* in “晴, *sunny*”) and a phonetic radical that provides the sound cue of the character's pronunciation (e.g., “青, /qing1/” in “晴, /qing2/”; Li et al., 1992; Li F., 2005).

Radical are components that compose Chinese compound characters. In fact, many semantic radicals in the Chinese language are meaningful pictographic characters (Li et al., 1992; Li F., 2005). Radicals represent the semantic or phonetic information in compound characters (Shen and Ke, 2007; Chen et al., 2013). Thus, radicals are the major orthographic processing units in Chinese character recognition and reading development for native speakers (Chan and Nunes, 1998; Feldman and Siok, 1999; Taft et al., 1999; Ho et al., 2003; Wang et al., 2015), and in character learning and word reading for nonnative Chinese learners (Taft and Chung, 1999; Jin, 2003; Wang et al., 2004; Shen and Ke, 2007; Chen et al., 2013; Tong and Yip, 2015; Zhang et al., 2016). In the study by Wang et al. (2015), 73 native Chinese children were asked to choose the pictures that matched the meaning of 15 target semantic radicals (e.g. “犭”), the mean of scores was 9.24 (61.6%), indicating that even children in kindergarten have acquired some semantic radicals' meaning, and this ability, named “semantic radical awareness” in the children at time 1 could uniquely predict both word reading and word writing at time 2 (a year later) with age, nonverbal reasoning and time 1 performance controlled.

Semantic radical awareness can help readers disambiguate homophones, which are abundant in the Chinese language. With approximately 400 possible syllables (or approximately 1,200 when tones are considered) representing thousands of characters, homophones are more prevalent in Chinese than in most other languages (Shu and Anderson, 1997). Among the vast number of homophones, many characters containing a common phonetic radical share the same pronunciation. For instance, three homophones “清, /qing1/, *clear, cleanup*”, “鲭, /qing1/, *mackerel*”, “蜻, /qing1/, *dragonfly*” share the same phonetic radical “青, /qing1/”. In addition, some characters “晴, /qing2/, *sunny*”, “请, /qing3/, *invite or request*”, and “睛, /jing1/, *eye*”, share the same phonetic radical but may have slightly different pronunciations. These homophones may cause difficulties and ambiguities in reading comprehension. Semantic radicals help readers disambiguate these homophones. In the aforementioned instance, the semantic radicals “氵, *water*”, “鱼, *fish*”, “虫, *insect*”, “日, *sun*”, “讠, *speech*” and “目, *eye*” can differentiate the meanings of those characters or provide the semantic connection between the radicals and the characters, such as water (“氵”) can clean up (“清”) something, and mackerel (“鲭”) is a type of fish (“鱼”). Shu and Anderson (1997) posited that beginning in the third grade, Chinese elementary children are aware of the relationship between the semantic radicals and the meaning of characters, and this ability can help them distinguish homophones.

Previous research has also found a *semantic radical bias* in character decoding and reading for nonnative Chinese learners (Williams and Bever, 2010; Tong and Yip, 2015). In a picture-character mapping task (Tong and Yip, 2015), 84 CFL learners were asked to choose among five logographic patterns that could best represent the picture for each item under three conditions: with no descriptive cue (e.g., a picture of “*bridge*” was visually presented followed by five logographic patterns: A. 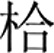, B. 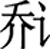, C. 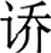, D. 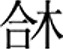, E. 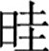), with a semantic cue (briefly describe the relationship between the semantic radical and the target character, e.g., “*Bridges were made of wood in ancient times in China*”), and with a phonetic cue (the sound of the target character was presented, e.g., /qiao2/). The results showed a strong preference of CFL learners for semantic radicals by choosing pseudo characters composed of semantic radicals in their correct positions (e.g., 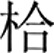) over those composed of phonetic radicals in their correct positions (e.g., 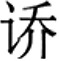) under both the no cue and the semantic cue conditions. Moreover, the predictions of semantic and phonetic radical sensitivity in word reading demonstrated that semantic radicals play a more important role than phonetic radicals in character learning for CFL learners (Tong and Yip, 2015).

### Development of semantic radical awareness in chinese nonnative speakers

As we mentioned, the structure of a SPC character is distinct with two different function radicals: semantic radical and phonetic radical. Wherein, the semantic radicals appear in many SPC characters with salient features of high combinability, transparent meaning category and fixed positions. In 514 common components in modern Chinese, a number of semantic radicals are high-combinability indexing components, such as “口, 日, 木, 氵, 扌, 艹” composing at least 167 characters (Specification of Common Modern Chinese Character Components and Component Names, 2009). A SPC family of semantic radical includes all characters sharing the same radical that provides a meaning category or relative domain. Generally, radical families are large with 15 members on average (Shu et al., 2003). Thus, the high repetition rates of these semantic radicals boost sensitivity and familiarity of semantic radicals for CFL learners in their SPC character learning. Besides, plenty of semantic radicals have quite stabilized positions in their SPC family. Most semantic radicals always occupy the left side of characters, and this type of SPC characters occurs 67.39% in SPC characters, such as “扌, *hand*.” Semantic radicals in 10.5% of SPC characters always appear on the top, such as “艹, *grass*” (Li et al., 1992). The position stability of most semantic radicals makes them identifiable in character recognition. On the other hand, semantic radicals provide character's meaning categories or domains. There are approximately 87% transparent (i.e., the meaning of the character is directly related to the meaning of its radical, e.g., character “瞧*, look*” is related to the radical “目, *eye*”) and semitransparent (i.e., the meaning of the character is indirectly related to the meaning of its radical, e.g., character “刻, *carve*” is related to the radical “刂, *knife*”) characters (Kang, 1993). Taken together, the characteristics of high combinability, stabilized positions and meaning support or semantic transparency of semantic radicals would count as regularities in SPC structures. So when learners pay attention to a semantic radical which includes several familiar characters (e.g., “眼, *eye*”, “盯, *stare at*”, “眨, *blink*”, “睡, *sleep*”, “眯, *take a nap*”), they may conclude a meaning category through semantic similarity in all SPC members in the radical family (e.g., semantic radical “目” is related to eye or the movement of eyes). So the CFL learners may acquire semantic radical categories when they have certain vocabulary and pay more their attention to these semantic radicals' characteristics, even though they are not explicitly taught by teachers from Chinese classes.

For nonnative speakers, Li R. (2005) developed a “meaning relatedness” task in which participants were asked to select a character from three options (e.g., A. 治, B. 提, C. 但) that have a related meaning to the target character (e.g., “抬”) to explore their semantic radical awareness. The results showed that CFL learners who had 7–10 months of Chinese learning experience were able to use semantic radical strategies (correct answer: B. 提). However, it remains unclear whether CFL learners can use radical knowledge to guess the meanings of unknown characters in a reading context. In another study, Shen and Ke (2007) argued that semantic radical perception, radical knowledge and radical knowledge application skills of alphabetic readers and Chinese learners do not develop synchronously across learning levels. Despite very limited knowledge of Chinese radicals with 1 month of learning time, CFL learners could still decompose compound characters into radical units (54.41%), and after 1 year of learning, this radical perception ability showed rapid growth, with a mean accuracy rate of 73.17%. However, learners need three full years to reach a higher level (72.45%) using semantic radical knowledge to learn new characters, and the radical knowledge task accuracy was only 70.95% even when the materials included 40 semantic radicals from a list of 100 high-frequency radicals (Shen and Ke, 2007). Taken together, semantic radical awareness does not only concern the perception of semantic radicals but also emphasizes the awareness of utilizing semantic radical knowledge to infer the meaning of entire characters. Consequently, we hypothesized that there are two main parts composing the development of the semantic radical awareness, that is, the ability to conclude the semantic category of one radical from their learning experience and the ability to apply the semantic category in SPC characters learning.

Moreover, use of contextual information from reading is also needed when learners use the semantic category of a radical (e.g., “目, *eye*”) in lexical inference process of new characters it forms (e.g., “眶, *eye socket*” or “睃, *look askance at*”). Nagy et al. (1987) investigated incidental learning of word meanings from context during normal reading among American children, and suggested that if children are given texts they can comprehend, they will gain some knowledge about the meanings of some unfamiliar words. Although learning from context is more difficult in a second language, second-language readers have been shown to gain significant word knowledge simply from reading (Nagy, 1995). For Chinese native children, Tse et al. (2007) developed an integrative perceptual approach to teaching Chinese characters based on the phenomennographic theory of learning. The instructional process goes from whole to part, that is, character learning is anchored in text and the context is meaningful to the learners. In addition, new words generally become obstacles to reading comprehension when CFL learners have limited vocabulary. Because of the higher percentage of SPC characters in the later period of learning (Feng, 1998), more unfamiliar SPC characters would appear during reading. While awareness of semantic radical function could help readers accomplish semantic access at least to some extent by using the information about semantic radicals to guess the character meanings or their semantic categories, the context of the reading materials may provide other related information to promote the lexical access for SPC characters. For example, when the learners read phrase “热泪盈眶, *ones' eyes filled with tears*/ *cry one's eyes out*” with an unknown character “眶, *eye socket*”, they can also guess the character relates to “eye” by the radical “目, *eye*” and also by the character “泪, *tear*” appearing in the phrase. Therefore, not only the semantic radical, but also the context would be helpful to solve the reading obstacles. In the current study, we designed a sentence cloze task with all sentences providing medium level of contextual support based on the premise that CFL learners might also benefit from the sentence context to access a new character meaning.

In short, the integration of the implicit learning of semantic radical category and the utilization of contextual support from reading text may explain semantic radical awareness in Chinese characters learning for nonnative speakers. However, the ability of radical knowledge application in CFL learners became high (72.45%) when they had finished three full years of learning (Shen and Ke, 2007), although their scores still had not reached their highest performance. CFL beginners rarely use radicals in learning characters (Jiang and Zhao, 2001; Zhao and Jiang, 2002), thus an explicit teaching of semantic radical is needed for the nonnative Chinese learners to facilitate their semantic radical awareness.

### Semantic radical teaching methods for CFL learners

In early research, Taft and Chung (1999) taught beginning Chinese learners a set of 24 left-right-structured Chinese characters. The results indicated better semantic learning when the radicals were highlighted during the first presentation of characters to novice learners (Radicals Early). Compared with the Radicals Before group (which was told about radicals before seeing any characters), the Radicals Late group (which was told about radicals at the third presentation of characters) and the No Radicals group (which was told nothing about radicals at all), the learners in the Radicals Early group performed best both in immediate meaning recall and in delayed recall 1 week later (Taft and Chung, 1999). The findings suggested that explicit knowledge of the internal structure of Chinese characters could help beginners learn Chinese characters faster. Wang et al. (2004) applied a short period of instruction teaching the function of the semantic orthographic components to 15 first-year Chinese learners, and the results showed that students used the semantic radical information to infer the meaning of previously unknown characters after explicit instruction on the meaning of the target radicals, and the learning effect for low-frequency radicals was significant. Research focusing on teaching Chinese using multimedia instructional software has demonstrated that radical teaching is an effective strategy (Jin, 2003; Hao et al., 2010; Chen et al., 2013). According to Jin (2003), English-speaking learners of Chinese performed best in a recall task on 36 Chinese characters that were displayed on a computer with a radical presentation (i.e., the character origins and their semantic and phonetic components are displayed on the computer screen, e.g., “靶, /ba3/, *target*” followed by “革, *leather*” and “巴, /ba1/”). In comparison, students performed worse when characters were presented by stroke sequences and pronunciation (Pinyin). Using a radical-derived Chinese character e-learning platform in which learners could use either a semantic radical or a phonetic radical to learn a set of characters that are related derivations significantly enhanced the learners' orthographic awareness (Chen et al., 2013). In short, radical knowledge can be very useful information in learning Chinese characters for CFL learners, and teaching semantic radicals as a targeted strategy may provide more useful knowledge for learners to facilitate their semantic radical acquirement and characters learning.

However, Zhang et al. (2016) also found that without explicit teaching, beginning nonnative students of Chinese can implicitly use known semantic radicals to learn the meanings of new characters in a paired associate learning task. In that study, each character was presented with a novel picture, a verbal code (a short oral description of the character meaning) and a nonverbal code (the picture) that had addictive effects on the meanings recall of new characters. How could the CFL students learn the new characters even those were untaught SPC character? As we mentioned, for semantic radicals with high combinability, stabilized positions and semantic transparency, the CFL learners may be aware of semantic radical categories through these characteristics which appear in a semantic radical family they learned, then transfer the knowledge of semantic categories to learn other characters of the same semantic radical family. Thus, when the learners have reached a certain level of vocabulary, their Chinese experience would facilitate new characters learning. Woolfolk (2014) defined transfer: “Whenever something previously learned influences current learning or when solving an earlier problem affects how you solve a new problem, transfer has occurred.” (p. 383). Transfer may also occur for learning strategy if the strategy has been taught previously. In other words, when the students receive an effective strategy to learn several semantic radicals through an instruction, they can also flexibly apply the strategy to other semantic radicals learning.

### The present study

The present study attempted to investigate whether teaching semantic radical knowledge would improve CFL learners' ability to infer the meanings of unfamiliar Chinese characters in sentence reading. Specially two question were asked: (1) Can a short and intensive instruction of semantic radicals help CFL learners apply the taught radical knowledge to infer the meanings of new characters in sentence reading context? (2) Can CFL learners transfer the semantic radical strategy to infer the meanings of new characters that do not contain the taught semantic radicals? A sentence cloze task that requires students to choose an appropriate character that fits the sentence was administered before and after the instruction. Based on the finding and the development of the CFL learners' semantic radical awareness (Li R., 2005; Shen and Ke, 2007; Zhang et al., 2016), we hypothesized that students who were taught 12 target semantic radicals might transfer the semantic radical strategy from the instruction to learn characters containing other semantic radicals that they have not been taught. Thus, two experimental groups were selected to receive two counterbalanced interventions with two different sets of instructional materials. Both sets of materials were tested in the pre-test and post-test, the teaching materials of one group were used as the other group's transfer materials, and vice versa. Therefore, pre- and post-test gains in the teaching and transfer sets of the sentence cloze task would show the teaching effect and transfer effect of semantic radical teaching, respectively. The semantic radical teaching strategy emphasized showing the students how to establish a connection between the radical knowledge and the characters' meaning. The familiar characters that the students had already learned were used as teaching instances to express the semantic cues that the radicals provided to help them infer the meaning of entire characters, and based on this method, the students could try to guess the related meaning or the semantic category of other unfamiliar SPC characters that share the same radical. In other words, this method, called “old-for-new,” aimed to promote semantic radical awareness in CFL students and was encouraged for application in reading comprehension to overcome the barrier of new words that learners might see while reading.

A control group that engaged in “business-as-usual” also participated in the same pre-test and post-test. Considering the difficulty of sentence reading for beginning Chinese learners, we selected participants who had finished 1 year of Chinese study to ensure that they had steadily developed the ability of radical perception (Shen and Ke, 2007) and would have the basic vocabulary to understand simple sentences in the cloze test.

## Materials and methods

### Participants

This study was approved by the relevant Research Ethics committee of Beijing Normal University and was conducted in conformation with the relevant regulatory standards. Written informed consent was obtained from classroom teachers and all of the participants for their participation prior to data collection.

A total of 54 Vietnamese students (mean of age = 19.63, *SD* = 0.59, 53 female) majoring in Chinese Language from a university in Hanoi, Vietnam participated in this study. All of the students had Vietnamese as their home language, and none had prior Chinese learning experience before attending the university. They had more than 7 years of English learning experience. All of the participants were taught Chinese by Vietnamese teachers. The first year of their Chinese learning program consisted of reading-writing comprehensive courses, speaking and listening courses, 12 credit hours in all. There were thirteen 45-min sessions per week, and 30 weeks per academic year. All of them were taught with the main textbook entitled “*Han Yu Jiao Cheng*” (Yang, 1999). The textbook has six volumes and has total of 100 lessons with increasing difficulty. Only simplified versions of the characters were taught. The number of new words introduced in all six volumes of the textbook for the first year students was approximately 3,300. The participants took part in our study when they had finished the first 2 months of the fall semester in their second academic year. For the second year of their Chinese learning program, they received 4 courses of listening, speaking, reading and writing. They were taught with the main textbook entitled “*Qiao Liang*” (Chen, 1996) which is used for a practical intermediate Chinese course, and requires the learners to master at least 2,500 words.

The participants were recruited from four natural classes. Two classes were randomly assigned to two experimental groups that received an intervention of semantic radicals, each group had 18 students. Other 18 participants of the control group were selected from another two classes, all of them had 1 year of Chinese learning experience as well as the experimental groups' participants. The main reasons for selecting these participants for this study were the homogeneity of the Chinese literary levels and the fact that the learning environment could be controlled, whereas foreign students in China come from different countries with a variety of home languages and have different Chinese learning experiences. All of the students had the same Chinese learning background, the same learning duration, and similar Chinese proficiency levels.

Furthermore, the Vietnamese students in the present study have Vietnamese as their first language. Vietnamese is an isolating, monosyllabic and tonal language with six tones (Le et al., 2004), and the tones are used as phonemes since a change in tone indicates a change in meaning (Tang, 2007). Given that these characteristics in Vietnamese language are similar to Chinese language, Vietnamese writing system, however, is written as an adaptation of the Roman alphabet (e.g., chữ quốc ngữ). Thus, the Vietnamese students might be unfamiliar with the features of Chinese characters, such as strokes, stroke patterns and character's internal structure.

### Measures

#### Sentence cloze task

The participants were asked to choose the target character from four options to fill in the blank in a sentence. These options were four unfamiliar compound characters with the same phonetic radical and different semantic radicals. For example, the sentence “孩子一头扑进我的怀里, 眼泪打湿了我的衣___。” (*The child threw herself into my arms, and her tears moistened my ___*) was followed by four options: A. 襟 (*front of a garment*), B. 噤 (*keep silent*), C. 僸 (*brace oneself up*), D. 澿 (*water*). A was the correct answer. The participants received 1/0 point for a correct/incorrect answer and the maximum score was 48. Cronbach's alpha coefficients for the task in the pre-test and the post-test were 0.74 and 0.83, respectively.

The task consisted of 48 target compound characters consisting of 24 target semantic radicals. All of the target characters (Appendix A) had very low frequencies. The cumulative frequency was >0.93 in the Chinese Corpus of Center for Chinese Linguistics PKU^1^ of 10645 characters. All the target characters were confirmed by the teachers as untaught characters. All of the semantic radicals were productive and semantically transparent. The set of target radicals was selected from “Modern Chinese Dictionary” (Institute of Linguistics, Chinese Academy of Social Sciences, 2005) and the orthographic combinability (the number of characters that a semantic radical composes) were in the range of 18–438 characters (*M* = 111.33, *SD* = 95.43). Thirteen Chinese students were asked to rate the transparency of those 48 characters on a seven-point scale (from 1 = radical and character have no semantic relationship to 7 = radical and character have the most semantic relationship). The rating results showed that the 48 target characters were highly transparent (*M* = 5.67, *SD* = 0.68). For example, the target radical “衤/衣, *clothes*” formed two target characters—“襟, *front of a garment*” and “裘, *fur coat*.”

The 24 target radicals (composing 48 target characters) were divided into two sets: Cloze A and Cloze B, with 12 radicals and 24 characters in each set (Appendix A). Cloze A and B were used as the teaching materials for experimental group A and B respectively, and were also used as the transfer materials for experimental group B and A respectively to explore the different effects of mastery and transfer in the instruction. Thus, the two sets of materials for teaching and transfer for experimental group A and experimental group B were counterbalanced. It is important to ensure that the two sets of materials were equivalent in character characteristics, such as the combinability of target radicals, the frequency and transparency of the target characters. The *t*-test results showed that neither radical combinability nor character frequency were significantly different between the two sets of materials, *t*_radical combinability (11)_ = 1.82, *p* = 0.10; *t*_character frequency (23)_ = −0.79, *p* = 0.44. The two sets of materials were also not significantly different in transparency of character, *t*_character transparency (23)_ = 1.78, *p* = 0.09.

Forty-eight sentences were originally selected from the Chinese Corpus of Center for Chinese Linguistics PKU and were adapted to the participants' reading level. The sentences were simplified for vocabulary and grammar to ensure that all students with 1 year of Chinese learning experience could easily understand them. If there were any new words that the participants could not recognize, the researchers would directly translate them into Vietnamese to ensure that the participants could understand the meaning of the sentences, but no further explanation about the new words or sentences was provided. The length of the sentences ranged from 13 to 47 characters (*M* = 27.27 characters). Because the semantic cues of the target characters provided by the sentence context may vary, all of the sentences were assessed for contextual support by thirteen Chinese college students on a seven-point scale (from 1 = no support to 7 = strongest support). All of the sentences with four options, target characters and the meaning of target characters were represented. The students were asked to choose a rating to represent the strength of meaning support of the sentences that may help participants to choose the target characters to fill in the blanks. The contextual support of all of the sentences was medium, *M* = 4.67 (*SD* = 1.02). The two sets of materials were also not significantly different in the sentences' contextual support, *t*_contextual support (23)_ = 0.94, *p* = 0.36.

#### Chinese character recognition task

This task consisted of 50 Chinese characters selected from the “*Han Yu Jiao Cheng*” employed in the curricula in the first-year program at the selected university for teaching Chinese (Appendix B). These characters were rated as medium to difficult by the instructors. All of the participants were asked to write down the Pinyin form (the alphabetic script used to indicate the pronunciation of a character, which CFL learners generally study in the first 2 weeks of a Chinese language program) of each character and then use the character to form a compound word, a phrase, or a short sentence to determine that the student had completely mastered the character's meaning. If the character could not be recognized, the participants had to write down an “
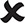
” symbol instead. For example, the Pinyin of character “趣” is “qù,” which can form the words “兴趣, *interest*,” “乐趣, *delight*,” or “趣味, *interest or delight*.” The participants received 1 point when they could provide the correct Pinyin form of the character and also could use the character to form a correct compound word (or phrases or short sentences). If they only finished one of these two requirements, they would get no point. The maximum score of the task was 50. Cronbach's alpha coefficient for the test was 0.91.

### Design and procedure

#### Pretests

All of the participants took the sentence cloze pre-test and engaged in the Chinese character recognition task. One week later, two experimental groups received a 90-min semantic radical teaching intervention during the regular Chinese lesson time. The experimental groups A and B learned the teaching material sets A and B, respectively. The control group continued their regular lessons. Immediately after the instruction, all of the students took the same sentence cloze post-test.

#### Semantic radical instruction

Twenty-four target semantic radicals in the sentence cloze task were used as teaching materials. The instructor was a trained researcher and a former Chinese lecturer at the university. The instructor used mostly Vietnamese language and partially Chinese language during the instruction.

The 90-min semantic radical lesson included (1) a brief introduction to compound characters and semantic radicals and (2) the method of using semantic radical knowledge to anticipate the meaning of an entire character.

(1) For the brief introduction to compound characters and semantic radicals, the instructor first wrote on the blackboard 4 types of Chinese character structure: pictographic, ideographic, associative compound and SPC. One example was given for each type.

Teacher: (Write down character 木 and read /mu4/.) This is a pictographic character, it was shaped from a tree. (Write down a little horizontal stroke on the character 木 to get new character 本 and read /ben3/.) This is an ideographic character, points to the root of the tree. (Write down character 林 and read /lin2/.) Putting together two and more single characters can create new compound characters. For example, this one is composed from two character 木, it means many trees will form a forest 林. This is an associative compound character. This kind of character in general is a combination of two meaningful single characters or radicals. Different from associative compounds, SPCs are combinations of two radicals with different functions, one for characters' meaning and one for pronunciation, they are named semantic radical and phonetic radical, respectively. (Write down characters 清, 晴, 请, 情.) More than 80% of Chinese characters are SPCs, such as these familiar characters you have learned, they are all composed of the same phonetic radical /qing1/. (Point to the radical 青 in four characters.) So they have the same or similar pronunciation. However, when we differentiate these characters, we need to bases on the semantic categories that different semantic radicals serve. Look at the character 请/qing3/, its semantic radical is 讠/yan2/. (Write down radical 讠 and its traditional form 言.) We can see a mouth 口/kou3/ on the bottom and some strokes above, it looks like some sound waves go out from the mouth, so the radical relates to speaking, talking, speech or language.

Students: (Listen and take notes.)

Teacher: On the other hand, 讠 appears in many characters you have learned, such as 说/shuo1/, 话/hua4/, 语/yu3/, 读/du2/. (Write down the characters.) We can see all of them are composed of the radical 讠, and their meaning relates to speaking, talking or speech. You may see some unfamiliar characters such as 训/xun4/, 讽/feng3/. (Write down the characters.) In general, they also relate to speech or spoken language.

**Figure 1:**
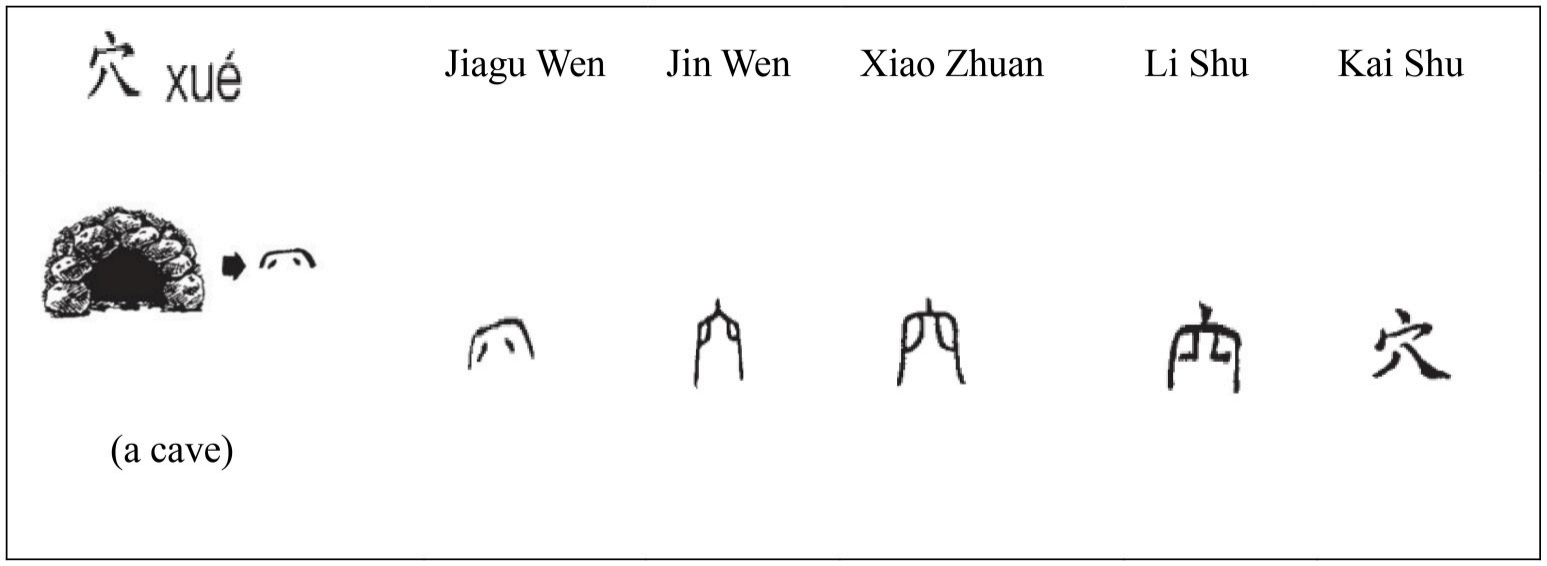
Example of semantic radical teaching materials (Li, 1999, p. 405).

(2) For the method of using semantic radical knowledge to anticipate the meaning of an entire character, the instructor first showed a picture of an object that carries the meaning of a target semantic radical and the transformed scripts (include Jiagu Wen -oracle script, Jin Wen-bronze script, Xiao Zhuan-small seal script, Li Shu-clerical script and Kai Shu -regular script) of the radical to help the students master the original meaning of that radical and understand that the semantic radical was primarily created by the symbolic change of the object. Second, the instructor introduced a common semantic category of the radical in contained characters from the modern Chinese vocabulary and then explained in detail several familiar characters' internal structure and their semantic construction from the knowledge of the target radical. For the instance of radical 穴 (Figure 1):

Teacher: (Show an A4 paper printing a small picture of a cave.) What do you see here?

Students: It looks like a cave (or a hole) of animals.

Teacher: Yes. It is a cave. Please look at the symbols near by the picture of a cave. (Show the varied transformations of its script from Jiagu Wen to Kai Shu.) The first signifying character drawing the shape of a cave liked this. After the evolution of the character, we have the character 穴/xue2/ that looks like it now. So the original meaning of 穴 was a cave. This is a single character, and also is a radical that can form some compound characters, and the characters made from this radical are in general related to a cave or a cavern.

Students: (Listen and take notes.)

Teacher: Can you tell me some characters composed from radical 穴 that you have been taught?

Students: Such as character 究 in word 研究, 窗 in 窗户 or 突 in 突然.

Teacher: Thank you. (Write down the characters) The character 究 that we are familiar with is a SPC character. It was composed of the phonetic radical 九/jiu3/and the semantic radical 穴 denotes the end of a cave, represented by thorough exploration or investigation. (Write down some words that includes the character 究.) Thus, we have the words and phrases 研究 (*research*) and 寻根究底 (*search to the root*) in Chinese. The character 窗 means a window. Do you know what the connection between a window 窗 and a cave 穴 is?

Students: (Puzzled).

Teacher: People in the old days considered that a window looked like a cave of a house where light and air can get in and out. (Write down character 突). This is an associative compound 突. (Point to the under radical 犬). What is this?

Teacher: /quan3/. It means a dog.

Students: Yes, it does. The character 突 indicates that a dog 犬 rushes out of the cave 穴, the movement is onrushing or sudden, thus we say the word 突然 (*suddenly*). So, we can see that, lots of characters which contain the radical 穴 have the meanings related to a cave.

The instructor also reminded the students to pay attention to the small differences between orthographically similar radicals such as “穴” and “宀” or “衤” and “礻.” The original meaning of the radicals and characters using in the instruction was referenced from some books written by Zhao (1988) and Li (1999).

In short, two experimental groups received the instruction of 12 semantic radicals including the knowledge about Chinese compound characters and semantic radicals' function; the introduction to semantic radicals' transformed scripts to help learners gain a deeper understanding of the original meaning of the semantic radicals; and the “old-for-new” method of using semantic radical knowledge to infer the meaning of an entire SPC character.

## Results

Table 1 presents the descriptive statistics for the performance of the three groups in the sentence cloze pre-test and the Chinese characters recognition task.

**Table 1:** Means, standard deviations of the accuracy rates in the Chinese character recognition task and the sentence cloze pre-test among three groups.

**Measures**	**Experimental group A (*N* = 18)**	**Experimental group B (*N* = 18)**	**Control group (*N* = 18)**
	***M* (*SD*)**	***M* (*SD*)**	***M* (*SD*)**
Chinese character recognition	0.39 (0.15)	0.39 (0.13)	0.40 (0.19)
**SENTENCE CLOZE PRE-TEST**			
Cloze A	0.54 (0.10)	0.50 (0.16)	0.49 (0.14)
Cloze B	0.50 (0.12)	0.48 (0.12)	0.45 (0.15)

The Chinese character recognition task was conducted to ensure that the experimental groups and the control group had equivalent character knowledge before the short instruction. Between-subject ANOVA showed no significant difference among the three groups in the character recognition task, *F*_(2, 51)_ = 0.03, *p* = 0.97. Therefore, the character knowledge was regarded as comparable across the three groups prior to the intervention.

A 3 × 2 (Group [experimental group A, experimental group B, and control group] × Material [Cloze A, Cloze B]) repeated-measures analysis of variance was conducted on the sentence cloze pre-test data. The results showed that the main effects of Material *F*_(1, 51)_ = 3.44, *p* = 0.07, η^2^ = 0.06, and Group *F*_(2, 51)_ = 0.83, *p* = 0.44, η^2^ = 0.03 were not significant. The two-way interaction of Material and Group was also not significant, *F*_(2, 51)_ = 0.19, *p* = 0.83, η^2^ = 0.01. These results suggested that the Cloze A and Cloze B were equivalent, and the three groups (experimental group A, experimental group B and control group) were comparable on prior knowledge of semantic radicals.

As mentioned earlier, set A was used as the teaching material (trained) for experimental group A, and as the transfer material (untrained) for experimental group B, set B was the opposite. Cloze A for experimental group A and Cloze B for experimental group B were combined and coded as Trained material, while Cloze B for experimental group A and Cloze A for experimental group B were coded as Untrained material. Table 2 presents the descriptive statistics for the performance of the experimental groups and control group in the sentence cloze pre-test and post-test.

**Table 2:** Means, standard deviations of the accuracy rates in the sentence cloze pre-test, post-test for the experimental groups and the control group.

**Measures**	**Pre-test**	**Post-test**	***F***	**η^2^**
	***M* (*SD*)**	***M* (*SD*)**		
**EXPERIMENTAL GROUPS (*****N*** = **36)**				
Trained	0.51 (0.11)	0.86 (0.09)	265.40^***^	0.88
Untrained	0.50 (0.14)	0.58 (0.16)	11.83^**^	0.25
Control group (*N* = 18)	0.47 (0.14)	0.50 (0.14)	3.42	

** p < 0.01;

*** *p < 0.001. The Trained material were Cloze A for experimental group A and Cloze B for experimental group B, the Untrained material were Cloze B for experimental group A and Cloze A for experimental group B*.

A 2 × 2 (Time [pre-test, post-test] × Material [Trained, Untrained]) repeated-measures analysis of variance was conducted on the data of the experimental group. The results showed that the main effects of Time *F*_(1, 35)_ = 190.70, *p* < 0.001, η^2^ = 0.85, and Material *F*_(1, 35)_ = 42.07, *p* < 0.001, η^2^ = 0.55 were statistically significant. These results suggested a pre-post difference, trained-untrained difference in the ability to use semantic radical knowledge to infer new character meanings during sentence reading. The two-way interaction of Time and Material was also significant, *F*_(1, 35)_ = 72.87, *p* < 0.001, η^2^ = 0.68.

Simple effect analysis was conducted. For Trained material, the accuracy rates in the post-test was higher than in the pre-test for the experimental groups, *F*_(1, 35)_ = 265.40, *p* < 0.001, η^2^ = 0.88, suggesting a significant teaching effect. For Untrained material, the accuracy rates in the post-test was also higher than in the pre-test, *F*_(1, 35)_ = 11.83, *p* < 0.01, η^2^ = 0.25. Although the effect size was not as large as that for Trained material, it indicated a significant transfer effect for the experimental groups. In other words, these interventional effects were significant not only for the teaching materials but also for the transfer materials.

Besides, the simple effect analysis results also showed that there was no significant difference between the two sets of materials (Trained and Untrained) in the pre-test for experimental groups, *F*_(1, 35)_ = 0.30, *p* = 0.59, η^2^ = 0.01. Nonetheless, the performance of the experimental group in the post-test on Trained material was significantly higher than their performance on Untrained material, *F*_(1, 35)_ = 79.74, *p* < 0.001, η^2^ = 0.70. These results indicated that the teaching effect on Trained material was bigger than the transfer effect on Untrained material.

It should be noted that the pre- and post-test differences in the control group was not significant at all, *F*_(1, 17)_ = 3.42, *p* = 0.08, η^2^ = 0.17, indicating no practice effect in the pre- and post-tests. Therefore, the effects of teaching and transfer among the experimental groups were not likely caused by familiarity with the sentence cloze test due to repeated measures.

In conclusion, the results revealed that the pre-test and post-test score increase was significant for the experimental groups, but not for the control group. Furthermore, the experimental groups successfully transferred the semantic radical strategy to figure out the meanings of unfamiliar characters containing untrained semantic radicals.

## Discussion

In the present study, Vietnamese students who learned Chinese as a second language for 1 year and received a short instruction of semantic radical could effectively use their learned knowledge of radicals to infer the meaning of unfamiliar compound characters in sentence-reading context. Extending previous studies (Taft and Chung, 1999; Wang et al., 2004; Shen and Ke, 2007; Tong and Yip, 2015; Zhang et al., 2016), this study provides evidence that a short and intensive semantic radical intervention can improve CFL learners' ability to apply semantic radical strategies to infer new character meanings in sentence reading.

In addition to the direct effect of radical teaching, more importantly, this study showed that foreign adult Chinese learners could also effectively transfer these semantic radical strategies to figure out the meanings of unfamiliar characters containing semantic radicals that they had not been taught in sentence context. In the condition of no practice effect for the sentence cloze test, the experimental groups' accuracy rates in the post-test were significantly higher than in the pre-test for both teaching and transfer materials (*F*s ≤ 11.83, *p*s < 0.01, η^2^s ≥ 0.25). Semantic radical teaching, therefore, directly improves semantic radical awareness for the radicals being taught and enables transfer to radicals not being taught. The results demonstrated the indirect effect of radical teaching among the students. Radical teaching provides a promising way to help students gain insight into radical knowledge and conjecture characters' meanings.

### Direct teaching effect and indirect transfer effect of the “old-for-new” semantic radical teaching method

**Figure 2:**
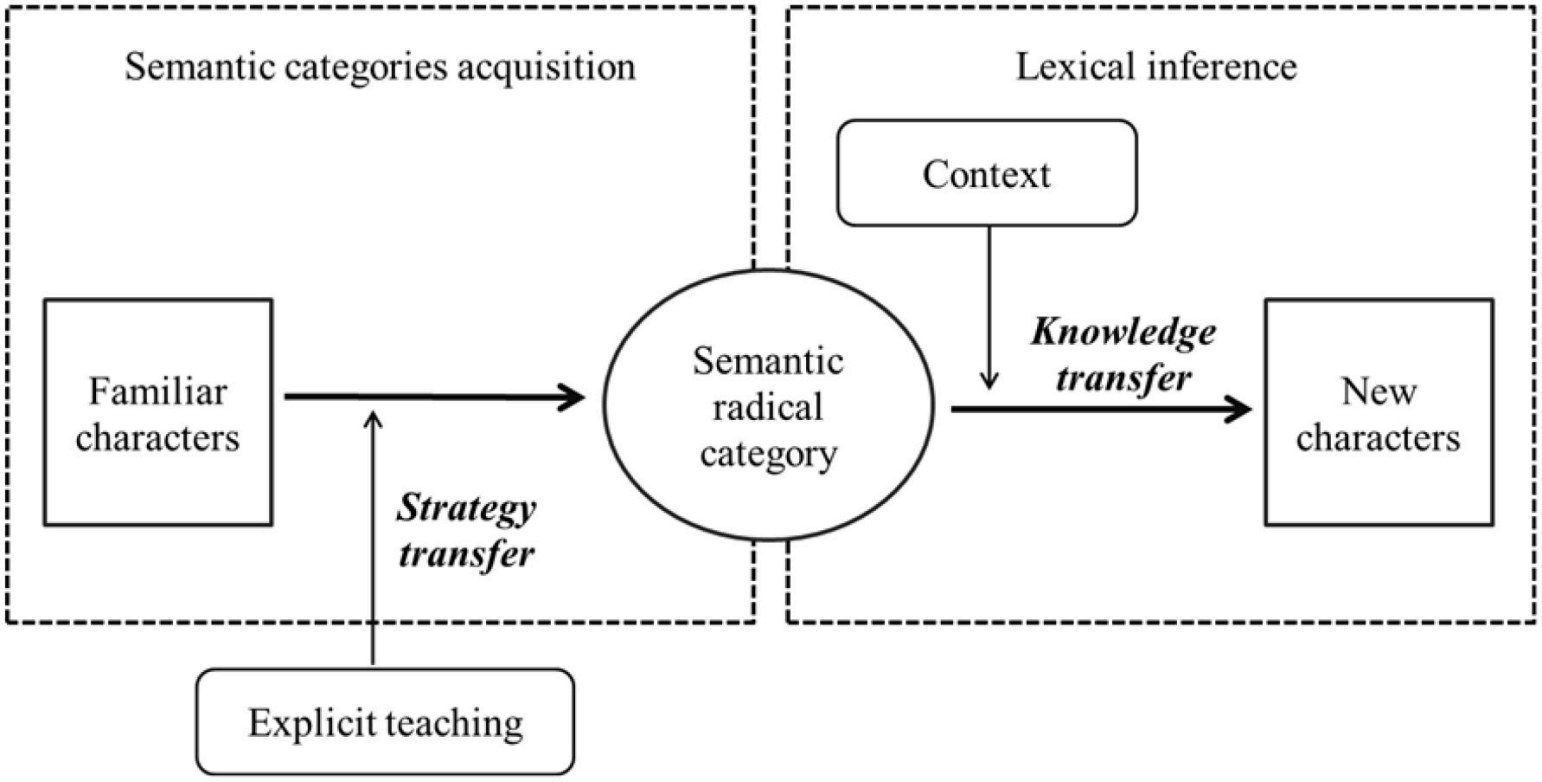
The framework of “Old-for-New” method in semantic radical teaching.

Figure 2 presents a framework to illustrate the “old-for-new” method in the current study. In the framework, *Semantic radical category* links *Familiar characters* (*OLD*) to *New characters* (*NEW*), and there are two major parts of the “old-for-new” processing: *semantic categories acquisition* and *lexical inference* in context. The *Semantic categories acquisition* concerns the ability of learners to conclude the semantic category of a radical through many familiar characters composing from that radical, and this ability is the first part of semantic radical awareness aforementioned. In contrast to previous studies (Shen and Ke, 2007), which suggested that CFL learners do not develop semantic radical awareness in the beginning stage of Chinese learning, our study showed that beginner Chinese language students already have adequate knowledge of semantic radicals. The accuracy rate in the pre-test of the sentence cloze task for 1-year learners was 0.49, exceeding the chance level (0.25). This result indicated that Vietnamese students who have 1-year of Chinese experience, had some insight into the semantic radical function of SPC characters, and were able to learn semantic category of several radicals that appeared in their Chinese learning through their implicit learning (Zhang et al., 2016). However, the semantic radical teaching applied in the present study made the implicit learning of semantic radical more explicit. Semantic radical teaching (*Explicit teaching* in the framework) could help the CFL learners determine whether or not the knowledge of semantic radical category they acquire from implicit learning is correct, and also enhance their ability to apply semantic radical knowledge to character meaning inference in sentence reading context. The supporting evidence is the high accuracies of performance on the teaching material (*M* = 0.86) after the instructions. Semantic radical teaching also provided a strategy of character learning that the learners could transfer to other materials that were not explicitly taught.

The *Strategy transfer* in the framework showed that, without explicit instruction, CFL learners can increasingly acquire radical categories through applying the strategy of semantic radical teaching. For CFL learners, transfer occurs when they use the semantic radical strategy to infer meaning categories from the teaching materials to other radicals not been taught, which is evident by the pre-post differences on the transfer materials for both experimental groups. It should be noted that, even when the ability to transfer semantic strategy to new materials develops, success of semantic category acquisition still depends on prior knowledge of semantic radicals. The transfer effect is built upon implicit learning of semantic radicals. In other words, the ability to acquire semantic radical category in Chinese learners depends on the implicit learning of frequent characteristics of semantic radicals, and the radical teaching facilitates both the direct learning effect of teaching materials and indirect effect by strategy transfer. Approximately 189 radicals are most frequently used in modern Chinese (Institute of Linguistics, Chinese Academy of Social Sciences, 2004), and it would be time consuming to introduce all of the radicals one by one in Chinese language teaching. However, when CFL students are aware of the function of semantic radicals in compound characters and have acquired a certain amount of Chinese vocabulary, they can use semantic radicals strategically to learn the meanings of new characters. Transfer may occur when learners have sufficient prior knowledge. The current results suggest that nonnative Chinese speakers can learn semantic radicals through the “old-for-new” teaching strategy when they have finished their first year of Chinese learning at a university.

The second major part of the “old-for-new” framework is *Lexical inference* which concerns the ability of learners to use the semantic category of radical that they acquire to infer the meaning of new characters composing from the radical. The *Knowledge transfer* in the framework indicates the knowledge application in lexical learning. In short, there are two kinds of transfer occurred: transfer of newly taught semantic learning strategy, and transfer of acquired semantic radical knowledge. Transfer is regarded as influence of previously learned materials on new materials (Woolfolk, 2014), thus we suggest that, the knowledge of a semantic radical (e.g., “目, *eye*”) that learners acquired may influence the semantic access of new characters that contain the radical (e.g., “睃, *look askance at*”). The transfer of knowledge, therefore, should be regarded as a spontaneous, initiative process in Chinese character learning, particularly for transparent SPC characters which have strong connection between semantic radical meanings and character meanings. However, new character learning is not only influenced by the transfer of prior knowledge but also supported by reading contexts.

The present study suggests that learners can use the semantic information to infer a new character's meaning, and this lexical inference process can be supported by contextual information. Context plays an important role in vocabulary learning (Nagy et al., 1987; Nagy, 1995). For the CFL beginners, given the limited vocabulary knowledge and vast homophones in Chinese, using sentences that provide some useful contextual information for inferring new characters' meanings is necessary. In other words, context is regarded as a supportable factor may help learners enhance their ability in semantic radical knowledge application. The cloze task used in the present study aimed to create a normal reading situation for lexical learning.

As we mentioned, the “meaning relatedness” task in the study by Li R. (2005), focused on the perception of learners about radicals' semantic function, but all the materials were characters, thus only lexical level was probed in the study. It remains unclear how students infer a character's meaning by using semantic radical strategies in normal reading. Unlike the “meaning relatedness” task, the current study demonstrated that beginning Chinese learners with 1 year of Chinese learning could differentiate compound characters with different semantic radicals and choose the appropriate characters for the blanks in sentences. In addition, compared to the study by Tse et al. (2007) using passage context for characters learning, the present study did not directly explore the role of context in new SPC characters learning, because CFL learners have limited oral vocabulary in Chinese. However, when Vietnamese learners reach the second year, they will have acquired at least 2,500 words as a requirement for second-year program's textbook (Chen, 1996), thus the learners could use some useful information from the sentences to understand the meanings of target characters so that they can choose a correct character that fits the meaning of whole sentences. The sentences in the current study provided medium to high level of contextual support to help learners easily comprehend the new character meanings.

In short, the process of “old-for-new” teaching strategy not only goes from whole to part (familiar characters to sub-lexical) but also works from part to whole (sub-lexical to unfamiliar characters). The radical teaching strategy has shown effectiveness for beginning Vietnamese learners who had 1 year of Chinese learning, so the “old-for-new” radical teaching is an effective strategy in CFL program and is suitable for beginning learners after a year of Chinese experience.

### The promise of morphology-based instruction of semantic radicals

In addition to the role of semantic categories acquisition and lexical inference in radical teaching, the “old-for-new” method worked effectively due to the brief instruction of radicals' original meaning and semantic function. First, the original meaning of a radical showed in a picture in teaching could help students memorize that radical's semantic category more accurately and deeply. In general, most semantic radicals were created from pictogram characters with simplified patterns and could stand alone as single characters (Li F., 2005). In modern Chinese, however, some semantic radicals have a different meaning from their pictogram pattern. For instance, “页” means “*page*” when it is learned as a single character in modern Chinese, but the original meaning of the radical “页” is “*head*”, and almost all of the characters it forms (e.g., “额, *forehead*”, “颊, *cheek*”) relate to the head of a man or a creature. In other words, the common meaning of the character “页” differs from its semantic category when it plays a radical role in many compound characters, even though the meaning cue it provided to understand the entire characters is transparent. If learners did not know the exact original meaning of “页,” how could they build a connection between “页, *page*”, and “额, *forehead*”, or “颊, *cheek*”? In another example, the radicals “礻” and “衤” look alike but have different meanings; “礻” is a sacrificial altar, and “衤” means clothes. When students can identify them through pictures of their original meanings, they might make fewer mistakes in recognition of characters that are orthographically similar (e.g., “祛, *dispel*” and “袪, *cuff of a sleeve*”). Therefore, the picture shown in the radical teaching did confirm the semantic categories of the radicals via their first meaning expressed in pictogram form and changing patterns so that the learners had more reliable and usable information about radical knowledge.

Second, semantic radical teaching includes not only the introduction of radical meaning but also focuses on establishing the relationship between radicals and characters. Semantic radical knowledge provides a clue to characters' meaning. In a few situations, such as “口, *mouth*” in “嘴, *mouth*” and “见, *see*” in “视, *watch*,” the radicals and character meanings are the same, but most radicals do not represent the exact meanings of characters. In general, radicals express the semantic category or domain of a character. Many characters do not maintain a meaning connection with their radicals; those irregular cases are called opaque characters (Shu and Anderson, 1997), which account for 13.26% of modern Chinese (Li et al., 1992). For example, the character “给, *give/for*” has no relationship with its constitute radical “纟, *silk yarn*”; thus radical knowledge does not provide any reliable guide to guess the character meaning. CFL learners, particularly beginners, are familiar with mechanical memorization in studying characters (Shi and Wan, 1998; Jiang and Zhao, 2001; Zhao and Jiang, 2002); thus the Chinese language, with its logographic writing system, is always a significant challenge for them. Even an awareness of radicals' existence can help learners crack a character's internal configuration into smaller units so they can memorize characters in an easier way (Nguyen et al., 2016), but it is not enough for the study of the Chinese writing system. In the current study, we gave learners a meaningful explanation about how a character was created and the contributed meaning of the radicals composed by it. For example, the character “忍, /ren3/, *to bear with*,” that is, the edge of a knife (“刃, /ren4/”) put on a heart (“心”), even it is very painful, but the heart still bears with (“忍”) the pain (Gu, 2008, p. 612). We guided the students to develop insight into the meaning of all of the internal units of a character to help them understand the character more deeply through the semantic radical information and their prior knowledge. We therefore encouraged the learners to seek the story of each character they learned and to enjoy learning the Chinese characters.

### Future directions and conclusion

The present study suggests that the “old-for-new” strategy is effective for promoting semantic radical awareness for transparent characters. As described above, opaque compound characters might become an interference factor in compound character learning for nonnative speakers. It remains to be investigated whether the semantic radical teaching strategy works effectively for opaque characters. A limitation should be taken into account is that the present study did not follow up the effectiveness of the instructional intervention after a month or in longer time later, the sample size was quite small, and the instruction was teacher directed. Moreover, more background measures about the Chinese reading ability of CFL learners (e.g., sentence reading comprehension and vocabulary) in areas other than character recognition should be taken into account in further studies.

To summarize, a semantic radical teaching strategy called the “old-for-new” method in the present study demonstrates an effective approach to improve the application of semantic radical knowledge to figure out the meanings of new characters in sentences reading among CFL learners. Not only the direct effect of teaching but also the indirect effect of transfer of the semantic radical strategy were found. The results underscore the value of explicit teaching of function and regularity of semantic radicals to integrate orthographic learning, incidental word learning and reading comprehension for Chinese as foreign language learners.

## Author contributions

Conception and design of the study: TPN, HL, and XW. Acquisition, analysis, and interpretation of data: TPN, JZ, HL, and YC. Drafting the work and revising it critically for important intellectual content: TPN, JZ, and YC.

### Conflict of interest statement

The authors declare that the research was conducted in the absence of any commercial or financial relationships that could be construed as a potential conflict of interest. The reviewer, WH, and handling Editor declared their shared affiliation.

## Footnotes

^1^*Centre for Chinese Linguistics*. Peking University. Available online at: http://ccl.pku.edu.cn:8080/ccl_corpus/.
