# Supplementary material for: Teaching Semantic Radicals Facilitates Inferring New Character Meaning in Sentence Reading for Nonnative Chinese Speakers
Source: Front Psychol. 2017 Oct 23;8:1846. doi: 10.3389/fpsyg.2017.01846 (PMC5660119; doi:10.3389/fpsyg.2017.01846)
Supplement: Supplementary file 1 [file Table1.docx]

**Appendix A.** Materials in the sentence cloze task and semantic radical teaching

| No. | Radical | Cloze | Character | No. | Radical | Cloze | Character |
| --- | --- | --- | --- | --- | --- | --- | --- |
| 1 | 心 | A | 恚 | 25 | 革 | A | 靶 |
| 2 |  |  | 怠 | 26 |  |  | 鞘 |
| 3 | 足 | A | 跬 | 27 | 弓 | A | 弩 |
| 4 |  |  | 跋 | 28 |  |  | 弦 |
| 5 | 饣 | A | 饴 | 29 | 酉 | A | 酣 |
| 6 |  |  | 馊 | 30 |  |  | 醮 |
| 7 | 钅 | A | 锷 | 31 | 纟 | A | 缣 |
| 8 |  |  | 锼 | 32 |  |  | 绫 |
| 9 | 艹 | A | 苔 | 33 | 皿 | A | 盅 |
| 10 |  |  | 荚 | 34 |  |  | 盂 |
| 11 | 虫 | A | 蜱 | 35 | 辶 | A | 遨 |
| 12 |  |  | 蝉 | 36 |  |  | 迢迢 |
| 13 | 犭 | B | 獓 | 37 | 囗 | B | 圃 |
| 14 |  |  | 獚 | 38 |  |  | 囩 |
| 15 | 疒 | B | 瘟 | 39 | 彳 | B | 徜徉 |
| 16 |  |  | 癖 | 40 |  |  | 徇 |
| 17 | 衤 | B | 襟 | 41 | 穴 | B | 窨 |
| 18 |  |  | 裘 | 42 |  |  | 窘 |
| 19 | 土 | B | 埂 | 43 | 页 | B | 颌 |
| 20 |  |  | 垟 | 44 |  |  | 颏 |
| 21 | 鸟 | B | 鹏 | 45 | 礻 | B | 祓 |
| 22 |  |  | 鹩 | 46 |  |  | 祛 |
| 23 | 目 | B | 睃 | 47 | 瓦 | B | 甓 |
| 24 |  |  | 眶 | 48 |  |  | 瓮 |

**Appendix B.** Materials in the Chinese character recognition task

| 1 | 毛 | 11 | 未 | 21 | 稀 | 31 | 截 | 41 | 殊 |
| --- | --- | --- | --- | --- | --- | --- | --- | --- | --- |
| 2 | 表 | 12 | 荣 | 22 | 忧 | 32 | 滋 | 42 | 蛮 |
| 3 | 肉 | 13 | 废 | 23 | 威 | 33 | 堪 | 43 | 雀 |
| 4 | 休 | 14 | 盼 | 24 | 愚 | 34 | 繁 | 44 | 寝 |
| 5 | 圆 | 15 | 遗 | 25 | 冻 | 35 | 倦 | 45 | 恕 |
| 6 | 趣 | 16 | 悲 | 26 | 怨 | 36 | 弘 | 46 | 毫 |
| 7 | 原 | 17 | 夸 | 27 | 寡 | 37 | 拢 | 47 | 僻 |
| 8 | 俗 | 18 | 缘 | 28 | 略 | 38 | 疏 | 48 | 缚 |
| 9 | 富 | 19 | 瞒 | 29 | 滞 | 39 | 剖 | 49 | 馨 |
| 10 | 签 | 20 | 残 | 30 | 钞 | 40 | 锐 | 50 | 怠 |
